# ZIBGLMM: Zero-inflated bivariate generalized linear mixed model for meta-analysis with double-zero-event studies

**DOI:** 10.1017/rsm.2024.4

**Published:** 2025-03-21

**Authors:** Lu Li, Lifeng Lin, Joseph C. Cappelleri, Haitao Chu, Yong Chen

**Affiliations:** 1 Center for Health Analytics and Synthesis of Evidence, Perelman School of Medicine, University of Pennsylvania, Philadelphia, PA, USA; 2 Applied Mathematics and Computational Science Graduate Program, University of Pennsylvania, Philadelphia, PA, USA; 3 Department of Epidemiology and Biostatistics, University of Arizona, Tucson, AZ, USA; 4 Statistical Research and Data Science Center, Pfizer Inc, New York, NY, USA; 5 Division of Biostatistics and Health Data Science, University of Minnesota Twin Cities, Minneapolis, MN, USA

**Keywords:** bivariate generalized linear mixed models, double-zero-event studies, generalized linear mixed models, meta-analysis, zero inflation

## Abstract

Double-zero-event studies (DZS) pose a challenge for accurately estimating the overall treatment effect in meta-analysis (MA). Current approaches, such as continuity correction or omission of DZS, are commonly employed, yet these ad hoc methods can yield biased conclusions. Although the standard bivariate generalized linear mixed model (BGLMM) can accommodate DZS, it fails to address the potential systemic differences between DZS and other studies. In this article, we propose a zero-inflated bivariate generalized linear mixed model (ZIBGLMM) to tackle this issue. This two-component finite mixture model includes zero inflation for a subpopulation with negligible or extremely low risk. We develop both frequentist and Bayesian versions of ZIBGLMM and examine its performance in estimating risk ratios against the BGLMM and conventional two-stage MA that excludes DZS. Through extensive simulation studies and real-world MA case studies, we demonstrate that ZIBGLMM outperforms the BGLMM and conventional two-stage MA that excludes DZS in estimating the true effect size with substantially less bias and comparable coverage probability.

## Highlights

**What is already known?**

- Double-zero-event studies (DZS) are challenging for meta-analysis (MA) due to division by zero errors.
- Current methods to address this include continuity correction or omitting DZS.
- These existing methods might produce biased conclusions.

**What is new?**

- The article introduces and discusses the rationale for proposing zero-inflated bivariate generalized linear mixed model (ZIBGLMM), a novel method for handling DZS in MA.
- The ZIBGLMM addresses issues by using a data-driven approach to identify subpopulations with extremely low risks and model population heterogeneity.

**Potential impact for Research Synthesis Methods readers**

- The proposed ZIBGLMM can potentially provide a more accurate estimation of effect sizes, especially in MA with excess DZS.
- Using a data-driven approach, ZIBGLMM provides a more robust solution to handle DZS compared to existing methods, which could lead to better conclusions in research synthesis.

## 1 Introduction

Meta-analysis (MA) serves as an important tool for synthesizing evidence from multiple studies, providing a systematic and comprehensive understanding of the overall treatment effect. However, double-zero-event studies (DZS)—those with no events in both arms—present critical statistical challenges, leading to potential numerical instability and bias in estimating treatment effects^1^ ^,^ ^2^. Such studies are particularly prevalent in fields associated with rare events, such as surgical complications or adverse drug reactions.^3^ ^–^ ^5^

Various strategies have been proposed for handling single-zero-event or double-zero-event studies in MA. For common effect size measures such as risk ratio (RR) and odds ratio (OR), there are divergent views on how to handle DZS. When there are zero events in one arm, the continuity correction with a fixed value of 0.5 is commonly added to each cell of a 2 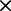 2 table for those studies.^1^ ^,^ ^6^ This approach allows the calculation of effect sizes such as RRs and ORs without encountering division by zero. If there are zero events in both arms, conventional practice omits these DZS from MAs of OR and RR, arguing that they contribute no information to the magnitude of the treatment effect.^1^ Bayesian approaches have also been proposed.^7^ ^,^ ^8^ However, even with non-informative priors, selecting the right prior distribution is crucial as it can significantly impact the analysis results, particularly in cases involving rare events.^9^ ^–^ ^11^

If risk difference is of interest, Tian et al.^12^ developed an exact inference procedure to synthesize evidence from DZS in an MA. Rücker et al.^13^ proposed to use the arcsine difference as a way to define treatment effects in MAs with double-zero studies. However, the limitation of the arcsine difference method is that the practical usage and interpretation of this difference in the arcsine scale is restricted.^14^

Notably, while continuity correction and omission are commonly used for handling DZS, these methods can lead to biased conclusions.^13^ Both the statistical significance and the direction of intervention effect can change after excluding DZS, as shown in both simulation studies and empirical data analyses.^14^ ^–^ ^16^ Although the continuity correction method can avoid computational errors, it usually could bias study estimates toward no difference and overestimate variances of study estimates.^4^ In addition, using different continuity corrections may result in different conclusions.^1^

**Figure 1:**
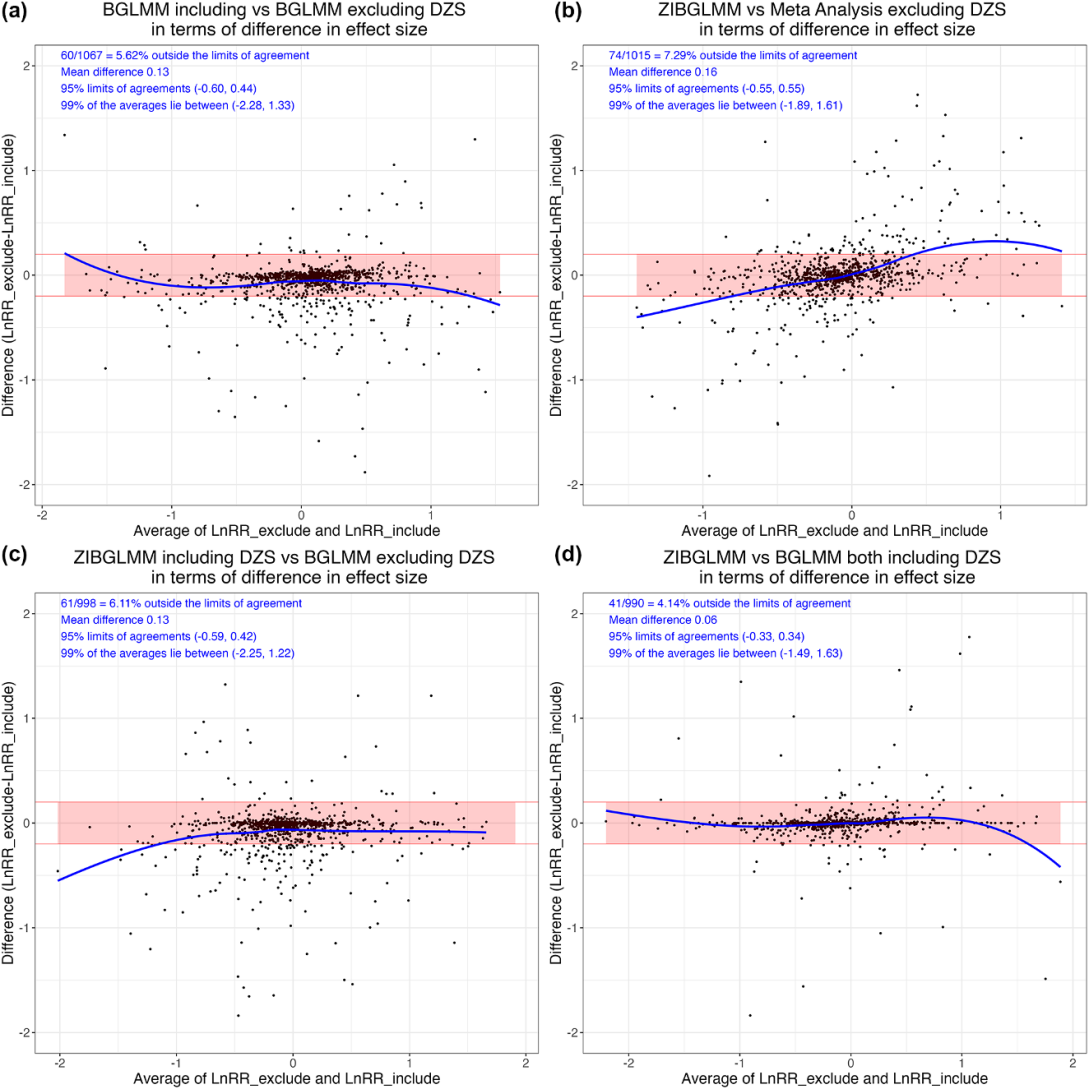
Estimated effect size differences using four methods, that is, bivariate generalized linear mixed model (BGLMM) including double-zero-event studies (DZS), BGLMM excluding DZS, conventional two-stage meta-analysis (MA) excluding DZS (MA), and zero-inflated bivariate generalized linear mixed model (ZIBGLMM) including DZS. In each subfigure, the *y*-axis is the difference in log risk ratios (RRs) between the two methods, and the *x*-axis is the average of the log RRs of the two methods being compared. Subfigure (a) contrasts the BGLMM with and without DZS. Subfigure (b) explores the difference in effect size between ZIBGLMM and MA, both excluding DZS. Subfigure (c) presents a similar comparison between ZIBGLMM including DZS and BGLMM excluding DZS. Subfigures (a–c) shed light on how the inclusion or exclusion of DZS significantly impacts effect sizes based on 1,111 Cochrane meta-analyses. Subfigure (d) provides a comparison between ZIBGLMM and BGLMM, both incorporating DZS. It illustrates a more concentrated distribution, signifying a smaller difference in log RR. Each subfigure displays the number of MAs lying outside the 95% limits of agreement, the mean difference between log RRs, 95% limits of agreements, and 99% range of the averages of log RRs at its upper left corner.

Excluding DZS is straightforward to implement but may lead to misleading inference. Böhning and Sangnawakij^17^ showed that double-zero studies do not contribute to the conditional log likelihood when using one-stage method using a Poisson regression model and a conditional binomial model to include the double-zero studies. However, as Xu et al.^3^ pointed out, this may not hold true for some other models such as the multilevel logistic regression model^18^ and beta-binomial model.^19^ In addition, excluding DZS does not fully utilize all the available evidence and can potentially lead to misleading inference if the excluded studies are systematically different from the included studies.^3^ ^,^ ^20^ ^–^ ^22^ Also, if the assumed underlying population event probabilities are not zero, DZS contain information for inference on the parameters such as the common OR in MA and can contribute to the estimation of treatment effects and thus cannot be left out in our analysis.^23^ Figure 1a illustrates how the effect sizes could change significantly depending on whether we include or exclude double-zero studies based on 1,111 MAs from Cochrane Database of Systematic Reviews (CDSR) (see details in Section 0.5).

Generalized linear mixed models (GLMMs) offer a flexible approach for modeling effect sizes and can incorporate information from DZS without ad hoc continuity correction.^18^ ^,^ ^24^ Bivariate generalized linear mixed models (BGLMMs) have been proposed to include random effects (REs) and potential correlation between treatment groups.^25^ ^–^ ^28^ These models can handle studies with zero events by specifying an appropriate link function and error distribution. For example, BGLMM has been applied to assess whether including DZS impacts the conclusions in a recent systematic review on prevention measures for preventing person-to-person transmission of COVID-19.^29^

Despite their utility, all the models mentioned above fail to address one potential key cause of DZS, that is, non-exchangeable heterogeneity in the population. DZS may occur if the study involves subpopulations with a negligible or extremely low probability of experiencing the event of interest. For instance, healthy subjects less than 65 years old only have negligible risks of experiencing hospitalization or death due to severe symptoms from COVID-19, compared to immunocompromised, unhealthy, or older subjects. The study populations with negligible or extremely low risks are fundamentally different from the remaining populations with low or moderate risks due to intrinsic differences in patient characteristics. Since the BGLMM assumes study populations are exchangeable, the above heterogeneity cannot be modeled properly with BGLMM.

To appropriately handle DZS and account for non-exchangeable heterogeneity in study populations, we propose a zero-inflated bivariate generalized linear mixed model (ZIBGLMM). Zero-inflated models have been commonly applied in other areas to model excess zero counts. Zero-inflated Poisson models have been applied to RE MA.^30^ Beisemann et al.^31^ compared three models (RE Poisson regression, RE zero-inflated Poisson regression, and binomial regression) to the standard methods in conjunction with different continuity corrections and different versions of beta-binomial regression.

Our article is the first to apply zero-inflated models in MAs to address non-exchangeable heterogeneity across study populations. It assumes that an

MA with a proportion of zero-event studies potentially contains two subpopulations: one with a near-zero risk and another with a higher risk. The ZIBGLMM can account for non-exchangeable heterogeneity as well as the correlation among studies in a data-driven fashion. Further, it can properly estimate the overall effect size by avoiding the biases in ad hoc solutions and can accurately infer the proportion of the low-risk population in each MA study.

Our contributions in this article are twofold. First, we introduce a novel method, ZIBGLMM, to incorporate DZS into MAs. Our model takes into account potential population heterogeneity and demonstrates its utility through both real-world and simulation studies. Second, we provide both frequentist and Bayesian implementations of the model. SAS and R implementations are publicly available in the GitHub repository.^32^

The rest of the article is organized as follows. Section 0.2 provides a motivating example that involves numerous DZS with various sample sizes ranging from 22 to 144 to demonstrate the rationale behind ZIBGLMM. Section 0.3 introduces the zero-inflated models and formulates both a frequentist and a Bayesian version of the model. Section 0.4 reanalyzes the example case study in Section 0.2 to demonstrate the clinical usefulness of the proposed method. Sections 0.5 and 0.6 present 1,111 real-world MAs from CDSR and 18,000 simulated MAs to compare the performance of various methods for estimating the RRs under various scenarios. Finally, Section 0.7 summarizes our key findings and limitations.

## 2 A motivating example

We explain the rationale behind the ZIBGLMM method using an example MA taken from the CDSR. The study investigates whether misoprostol could help prevent or treat excessive bleeding and reduce maternal deaths among women after birth.^33^ This review involves 19 studies, 5 of which are DZS. These DZS, with sample sizes ranging from 100 to 900, were excluded from the MA. Table 1 displays the specific data for these studies. The original analysis suggests that when comparing misoprostol using 600 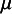 g misoprostol or more versus placebo or other uterotonics, the results for “maternal death or severe morbidity” are statistically nonsignificant (RR 1.67, 95% CI 0.80–3.45). In Section 0.4, we will revisit this case study to illustrate how including these DZS could yield different clinical conclusions.

**Table 1:** Motivation example study data^33^

	Experimental	Experimental	Control	Control	Posterior probability
Study	Number of	Sample	Sample	Number of	of belonging to the
ID	events	size	size	events	“structual”-zero
1	10	407	4	402	0
2	0	1,026	1	1,032	0
3	1	30	0	30	0
4	66	488	0	490	0
5	0	257	0	257	0.98
6	0	79	2	81	0
7	4	630	3	599	0
8	1	330	0	331	0
9	1	97	5	99	0
10	0	100	0	100	0.73
11	2	812	2	808	0
12	0	900	0	900	1
13	1	29	0	32	0
14	0	533	0	583	1
15	1	32	1	32	0
16	5	117	0	121	0
17	28	705	13	717	0
18	0	702	0	698	1
19	15	9,225	15	9,230	0

The rationale of the ZIBGLMM method is based on the observation that in an MA with a low overall event probability, the probability of encountering large studies with double-zero events should be low. For instance, given an event probability of 1%, the likelihood of a DZS with a sample size of 1,000 is approximately 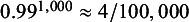 . Thus, it is improbable that these large DZS belong to the same population as the other studies included in the MA. This suggests that using the BGLMM method to incorporate all DZS and treating them as exchangeable with other studies may be inappropriate. Conversely, some smaller DZS may have double-zero events by chance, which means that completely excluding all DZS may not be the best approach either. The ZIBGLMM method addresses these issues by using a data-driven approach to identify the proportion of populations with extremely low risks and model population heterogeneity.

The proposed method is only intended to be used for MAs with a moderate to large size ( 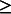 10 studies). We acknowledge that most of the real-world MAs are of smaller sizes.^34^ ^,^ ^35^ However, the sophistication of the proposed statistical model, which includes a bivariate outcome, a 2 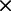 2 matrix of REs, and a mixture distribution, might lead to convergence issues in more realistic scenarios with fewer studies. Although this will potentially limit the broad impact of our method, we would like to avoid misleading conclusions when applying sophisticated models like ours to a limited number of studies.

## 3 Methods

### 3.1 Notation

Let 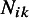 be the number of subjects, and let 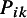 be the probability of an event for the 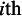 study ( 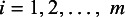 ), where 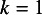 represents the treatment (or exposed) group and 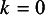 represents the control (or unexposed) group, respectively. Let 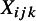 denote a Bernoulli random variable, with a value of 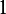 denoting an event and a value of 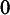 denoting a nonevent for the 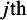 subject ( 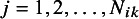 ) of the 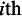 study in the 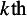 treatment group. Let 
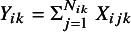
 be the total number of events in the 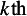 treatment group in the 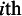 study. The event counts 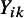 follow a binomial distribution, 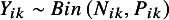 . Denote *n* as the total number of studies within an MA. The notations are summarized in Table 2.

**Table 2:** Descriptions of notation

Notation	Description
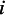	Index of study
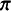	proportion of zero inflation
*k*	Index of treatment arm, 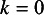 represents control group and 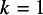 represents treatment group
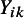	No. of events in the 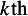 group in the 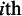 study
*n*	No. of studies in a meta-analysis
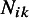	No. of subjects in the 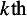 group in the 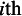 study
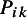	Probability of an event for the 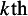 group in the 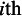 study
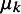	Fixed effects on the logit scale for the 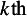 group across entire population
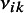	Random effects on the logit scale for the 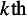 group in the 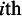 study

### 3.2 Zero-inflated models

The zero-inflated model proposed by Lambert^36^ has been widely applied to count data with excess zeros in various scientific fields such as industrial manufacturing,^36^ biomedical horticulture,^37^ and healthcare data.^38^ It is formulated as a mixture of a point mass at zero, which generates the excess zeros, and a count distribution that generates the remaining values. The zero-inflated models are particularly useful for data with a higher-than-expected number of zeros under standard count models. Besides commonly used zero-inflated Poisson models, Ghosh et al.^39^ extend the models to include a broad class of distributions (e.g., power series distributions). Böhning et al.^30^ apply the zero-inflated Poisson to RE MA. To the best of our knowledge, this work is the first application of zero-inflated models for handling DZS in the context of MAs with two arms using BGLMM.

Zero-inflated models, by design, segregate observed zeros into two distinct categories. The first category, often referred to as “structural” zeros, represents individuals who are not susceptible to a specific event, thereby having no chance of a positive count. The second category, known as “at-risk” or “chance” zeros, corresponds to a latent group of individuals who are at risk for an event but have a recorded count of zero. The zero-inflated binomial (ZIB) model can then be formulated as follows: 

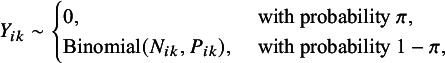

where 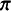 is the proportion of zero inflation, that is, the probability that a given subject belongs to the low-risk subpopulation.

For instance, in our study examining the number of people experiencing adverse events for probiotics, structural zeros might be indicative of patients in good health, resulting in experiencing no adverse events. Conversely, the at-risk zeros could represent patients who are more susceptible to adverse events, due to various circumstances, and experienced no adverse events by chance. Consequently, zero-inflated models can be interpreted as latent class models, where the classes are defined by these two types of zeros.^40^

Hall^37^ discusses classical statistical approaches that utilize the maximum likelihood estimation and the likelihood ratio (LR) test for zero-inflated Poisson regression. In the context of non-normal data, classical inference often relies on approximation theory, which is based on large sample sizes and may involve the application of nonstandard asymptotic theory.^41^ However, Ghosh et al.^39^ showed that the classical procedure does not perform well in estimating the zero-inflation probability when the sample size is finite, and the zero-inflation probability is close to unity, but the Bayesian estimates performed very well with respect to interval width and coverage probability.

### 3.3 Bivariate generalized linear mixed models

To estimate the effect sizes using conventional two-stage MA, one needs to estimate the event probabilities as 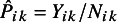 . For DZS, 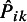 values are zero for both the treatment and control arms, creating numerical difficulty in estimating the effect sizes using RRs or ORs due to division by zero.

One approach to include DZS is to use the BGLMM method to directly model the event counts 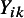 with binomial likelihoods instead of estimating the effect sizes of individual studies. The BGLMM can be specified as follows: let 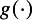 denote the link function that transforms event probabilities into linear forms. We have 

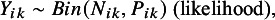




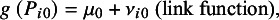




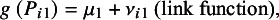




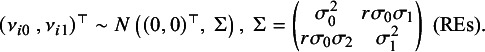

To implement the natural constraint of 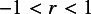 , one can use Fisher’s *z* transformation as 
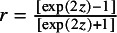
 .

We consider the logit link function for 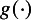 . The parameters 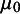 and 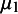 are fixed effects and represent the average risks in the control and treatment groups in the logit scale. Study-specific REs in the logit scale, 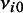 and 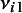 , are assumed to follow a bivariate normal distribution with a covariance matrix 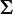 . The parameters 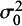 and 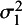 are between-study variances for the control and treatment groups due to heterogeneity, respectively, and *r* is the correlation between the two groups.

### 3.4 Zero-inflated bivariate generalized linear mixed models

One important limitation of the above BGLMM is its inability to account for excess DZS. This is because BGLMM ignores the intrinsic difference between DZS and the others by treating all studies as exchangeable, even if some studies may be conducted on different subpopulations.

To take into account the population heterogeneity, we introduce the ZIBGLMM method, which is a two-component finite mixture model. Specifically, we assume that the event probability in a certain study subpopulation, referred to as a “healthy population,” is extremely low, approximately equal to zero. In contrast, we assume that the other subpopulation, referred to as a “sicker population,” has a relatively high event probability. We denote 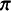 as the proportion of studies with healthy populations representing individuals who have approximately zero risk for the event of interest.

In the first stage, the ZIBGLMM combines two zero-generating processes for the number of events 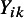 . The first process generates double zeros for both arms from extremely low-risk subpopulations. The second process is governed by a binomial distribution that generates the numbers of events, some of which may be zero by chance. The mixture is described as follows: 

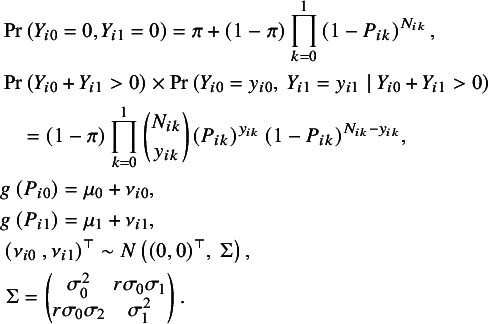



Of note, Dong et al.^42^ proposed a ZIB model for MA with sparse binary outcomes. However, they allowed for different proportions of zero-inflation for different treatment arms, which might not be the case for randomized control trials (RCTs), where the proportion of zero inflation for the two treatment arms should be the same. In contrast to their approach, we restrict the proportion of the healthy subpopulation to be equal across two treatment arms, which is more realistic given the setting of RCTs.

In this article, we focus on the overall treatment effect in the at-risk population measured by the marginal RR as suggested by McCullagh,^43^ ^,^ ^44^ which is defined as 
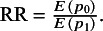
 The marginal event probabilities can be obtained through a well-established approximation formula: 
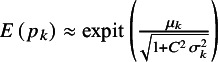
 for 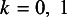 , with 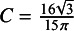 for the bivariate logit RE model.^45^

Our formulation of ZIBGLMM leverages zero-inflated models to account for subpopulations with an extremely low risk of experiencing the studied outcome. It uses a data-driven approach to capture the heterogeneity in the population, leading to improved model fitting and more reliable results in MAs.

### 3.5 Bayesian formulation

This section introduces a Bayesian formulation of the ZIBGLMM method. It is based on the Bayesian hierarchical model formulation of the RE MA model^46^ ^,^ ^47^ and the Bayesian zero-inflated models.^40^

Specifically, the Bayesian model can be formulated as follows, using the notations in Table 2: 

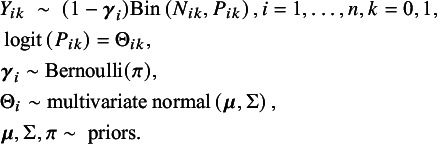

Here, 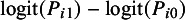 is the log OR. Adopting the same approach, an equivalent model on the RR replaces the logit function with the log function. That is, 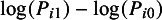 is the log RR. We employ a general procedure of data augmentation by including latent variables 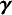 to the data *Y* such that 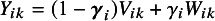 , where 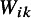 is the random variable with point mass distribution at 0, 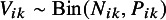 ,^48^ that is, 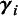 is a latent variable indicating whether the study is coming from the extremely low-risk population.

The Bayesian approach requires specifying prior distributions for 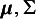 , and 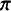 . Given the parametrization on logit scale, the event probabilities 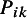 are forced to be between 0 and 1, and 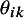 can range over the whole real line. A natural class of prior distributions for 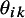 is the class of multivariate normal distributions. We assign the inverse-Wishart distribution, which is a multivariate analog of the gamma distribution, as the semi-conjugate prior distribution for the covariance matrix 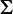 and the multivariate normal distribution as the prior for 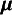 . We use weakly informative priors for the parameters of 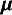 and 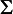 that are weakly centered around estimates derived from the observed data, that is, the population mean and covariance of 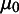 and 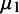 . These are commonly specified in normal hierarchical models.^46^ We use a beta prior for 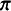 , that is, 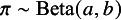 . Note that 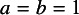 gives the uniform prior on 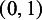 for 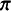 .

The joint posterior distribution is given by 

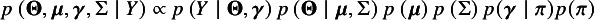

and 

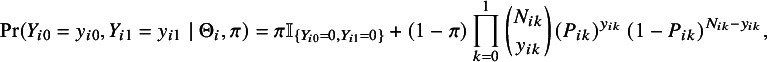

where 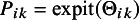 .

We use the Gibbs sampling approach^49^ for the estimation of our full posterior distribution of 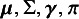 by sampling them from their full conditional distributions:

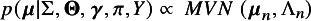
 , where 

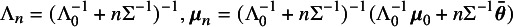

  and 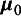 and 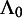 are the prior mean and variance of 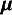 , respectively, and 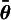 is the vector of treatment-arm-specific averages 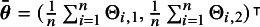 .

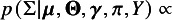
 inverse-Wishart 
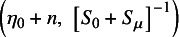
 , where 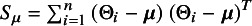 , and we take 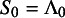 but only loosely center 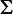 around this value by taking 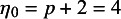 , where *p* is the number of treatment arms.

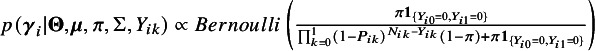
 , where 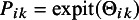 .

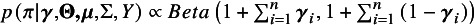
 .

We then use a Metropolis step^50^ for the estimation of the full posterior distribution of REs 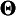 .^46^ ^,^ ^47^ The complete Metropolis–Hastings approximation algorithm is:

1. Sample 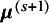 from 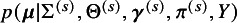 .
2. Sample 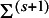 from 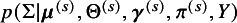 .
3. Sample 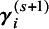 from 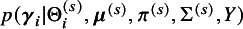 for 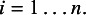

4. Sample 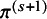 from 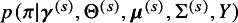 .
5. For each 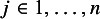 ,
  - sample 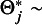 multivariate normal 
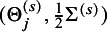
 ;
  - compute the acceptance rate 

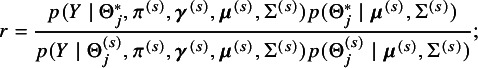

  - sample 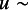 uniform 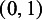 . Set 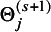 to 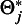 if 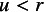 and to 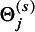 if 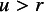 .

We used 100,000 iterations with thinning to keep every 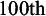 value and set the burn-in period to be 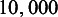 . We manually examined the trace plot and autocorrelation for a few example datasets to make sure the autocorrelation between the samples was low and the acceptance rate was around 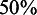 .

### 3.6 Implementation

The frequentist BGLMM and ZIBGLMM were implemented using PROC NLMIXED in SAS Studio 3.81 (Enterprise Edition). The REs were approximately integrated by the default adaptive Gaussian quadrature, and likelihood maximization used the conjugate gradient optimization algorithm. The Bayesian models were implemented using R version 4.2.2. In addition to our custom Bayesian Gibbs Sampler, we have implemented a version of the model using the RStan package using R version 4.2.2. This alternative provides a flexible framework, facilitating the customization and experimentation with various prior distributions. It is worth noting that our original model utilizes an inverse Wishart distribution for the priors and adapts the scale matrix dynamically during sampling, which can be complex to replicate in Stan. Consequently, the RStan implementation uses an LKJ correlation prior to the covariance matrix instead of the inverse Wishart distribution. The source SAS and R implementation code along with simulation and Cochrane datasets can be found in the GitHub repository.^32^

## 4 Case study

We apply our ZIBGLMM method to the MA of 19 studies conducted by Hofmeyr et al.,^33^ which involves 33,041 participants. This MA compares misoprostol use of greater than or equal to 600 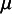 g dose versus placebo in terms of reducing maternal deaths or severe morbidity.^33^ The original analysis suggests that when comparing misoprostol use of greater than or equal to 600 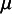 g versus placebo, the results for “maternal death or severe morbidity” are statistically nonsignificant (RR 1.67, 95% CI 0.80–3.45). In the original analysis, 5 of the 19 studies were DZS, with sample sizes ranging from 100 to 900, and were excluded from the analysis.

To evaluate the impact of using different strategies for handling the DZS on the results, we applied various methods to the same data as in Hofmeyr et al. When using the Mantel–Haenszel estimate^51^ of the risk difference, the estimated risk difference is 0.005 when including the DZS and changes to 0.006 when excluding the DZS. The estimated RR using the Mantel–Haenszel method is 2.84 (95% CI 2.03–3.99). Our Bayesian ZIBGLMM method yielded an RR of 2.98 (95% CI 1.97–4.62), which suggests a 198% increase in the risk of maternal death or severe morbidity associated with high dosage use of misoprostol compared with placebo. This conclusion is also consistent with the general recommendation of using the lowest effective dose of misoprostol to treat/prevent maternal bleeding after giving birth. Using the Bayesian BGLMM method yielded an RR of 2.81 (95% CI 1.78–4.37). This investigation underscores that neglecting DZS can lead to nonnegligible differences in the final estimated effects in MAs.

We note that the difference between the original analysis and the ZIBGLMM method might be due to the fact that they used conditional effects, while we focused on marginal effects.^52^ Nonetheless, the difference between the BGLMM and ZIBGLMM methods is 0.17, suggesting that accounting for DZS could lead to estimates that differ to a large degree.

The estimated proportion of structural zeros 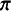 is 0.246. For every single study, the posterior probabilities of belonging to the “structural”-zero or “nonstructural”-zero study cluster are reported in Table 1. The estimated 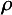 is 0.062, which suggests a slight positive correlation between the treatment and control outcomes within studies. The estimated variances are 0.079 and 0.061 for the treatment and control groups, respectively, which provide insights into the variability of the effect sizes within each group. The Akaike information criterion (AIC) of the frequentist version of the ZIBGLMM model is 1,756.3, and the AIC of the BGLMM model is 1,761.7. The deviance information criterion (DIC) of the Bayesian ZIBGLMM model is 979.02, and the DIC of the Bayesian BGLMM is 973.84.

## 5 Cochrane meta-analyses

**Figure 2:**
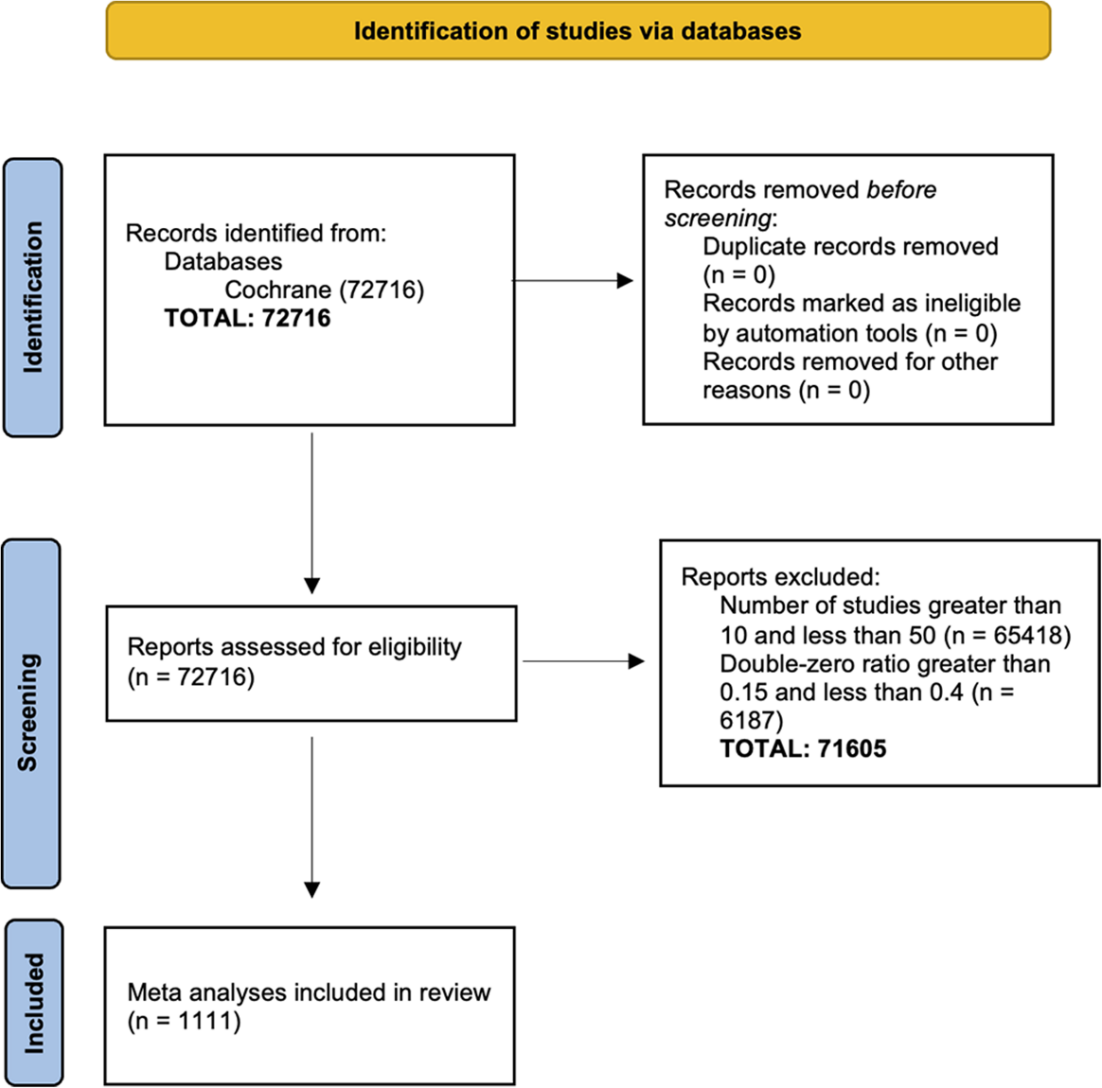
PRISMA plot of the 1,111 Cochrane Database of Systematic Reviews meta-analyses included in this article. We extracted 1,111 studies with sample sizes between 10 and 50 and with double-zero ratios between 0.15 and 0.4 from a total of 72,716 studies.

To evaluate the practical impacts of our ZIBGLMM method on a wide spectrum of MAs across different clinical domains, we conducted a meta-meta-analysis using a large number of datasets in the CDSR collected in our previous work.^53^ We identified 1,111 MAs, each containing between 15 and 50 studies (inclusive), with proportions of double-zero studies ranging from 0.15 to 0.4. Figure 2 shows the PRISMA (preferred reporting items for systematic reviews and aeta-analyses) flowchart detailing how we selected the 1,111 studies.^54^ Note that we do not know the true effect sizes for these real-world studies.

We meta-analyzed each dataset using a conventional two-stage method that excludes DZS, as well as both the frequentist and Bayesian versions of BGLMM and ZIBGLMM. When using the frequentist approach, 1,015 datasets converged for ZIBGLMM, while 1,084 datasets converged for BGLMM. The Bayesian BGLMM and ZIBGLMM successfully converged and provided the estimated effect sizes for all 1,111 studies.

To explore the impact of excluding DZS versus including DZS in an MA, we contrasted four methods based on the inclusion or exclusion of DZS, focusing on estimated effect size differences. The comparisons are listed below:

- BGLMM with DZS versus BGLMM without DZS: to investigate the impact of excluding DZS in one-stage method BGLMM.
- ZIBGLMM with DZS versus conventional two-stage MA without DZS: to investigate the impact of excluding DZS in conventional MA.
- ZIBGLMM with DZS versus BGLMM without DZS: to investigate the impact of including DZS versus not between two one-stage methods.
- ZIBGLMM versus BGLMM, both incorporating DZS: to investigate the impact of using different one-stage methods to incorporate DZS.

Specifically, we applied the Bland–Altman analysis^55^ to determine the difference in each comparison. The results are displayed in Figure 1. Notably, the mean effect size difference can reach up to 0.16 when comparing methods that differ in DZS inclusion (ZIBGLMM with DZS vs. MA without DZS). However, when both ZIBGLMM and BGLMM include DZS, this mean effect size difference narrowed down to 0.06. This suggests that omitting DZS can significantly alter effect size estimates.

Table 3 presents the results for further quantifying the impact of the exclusion of DZS and the methodological differences in both the magnitude and direction of log RRs. We detail the percentages of increase and decrease and document the count and proportion of pairs with different directions. The effect changed the direction if the RRs flipped, that is, log RR changing from “< 0” to “> 0.” The significance is flipped if the *p*-value transitioned from nonsignificant to significant, using a 0.05 threshold. We used the middle 99% of the RRs to leave out outliers.

**Table 3:** Summary of the comparative analysis of different methods in Figure 1

	Subfigure (a)	Subfigure (b)	Subfigure (c)	Subfigure (d)
	BGLMM w/	ZIBGLMM w/	ZIBGLMM	ZIBGLMM
	DZS vs.	DZS vs. two-stage	w/DZS vs.	w/DZS vs.
Subfigure	BGLMM w/o	meta-analysis w/o	BGLMM w/o	BGLMM w/
Methods being compared	DZS	DZS	DZS	DZS
No. of meta-analyses	1,067	1,015	998	990
Mean absolute difference in log RRs 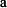	0.13	0.16	0.13	0.06
No. of meta-analyses with direction changes 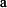	98 (9.29%)	130 (12.96%)	101 (10.22%)	37 (3.78%)
No. of meta-analyses with significance changes 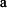	74 (7.01%)	108 (10.77%)	79 (8.00%)	51 (5.20%)
95% limit of agreements of log RRs	( 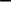 0.60, 0.44)	( 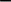 0.55, 0.55)	( 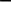 0.59, 0.42)	( 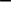 0.33, 0.34)
No. of meta-analyses with RRs increased 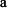	324 (30.71%)	465 (46.36%)	278 (28.14%)	428 (43.67%)
No. of meta-analyses with RRs decreased 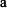	731 (69.29%)	533 (53.14%)	708 (71.66%)	466 (47.55%)

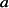
 Denotes using the middle 99% of data to exclude extreme values.

The highest percentage of direction change is observed in comparison B (ZIBGLMM with DZS vs. MA without DZS) at 12.96%, while the lowest percentage is observed in comparison D (ZIBGLMM vs. BGLMM both including DZS) at 3.78%. This indicates excluding DZS can lead to changes in effect directions. Comparison B (ZIBGLMM with DZS vs. MA without DZS) records the highest percentage of significance flip at 10.77%, with comparison D (ZIBGLMM vs. BGLMM both including DZS) having the lowest at 5.20%. The findings suggest that excluding DZS could lead to significant changes, that is, difference with a magnitude of greater than 0.1, in estimated effect sizes in MA. This finding is consistent across various methods, including conventional two-stage MA that excludes DZS, BGLMM, and ZIBGLMM.

**Figure 3:**
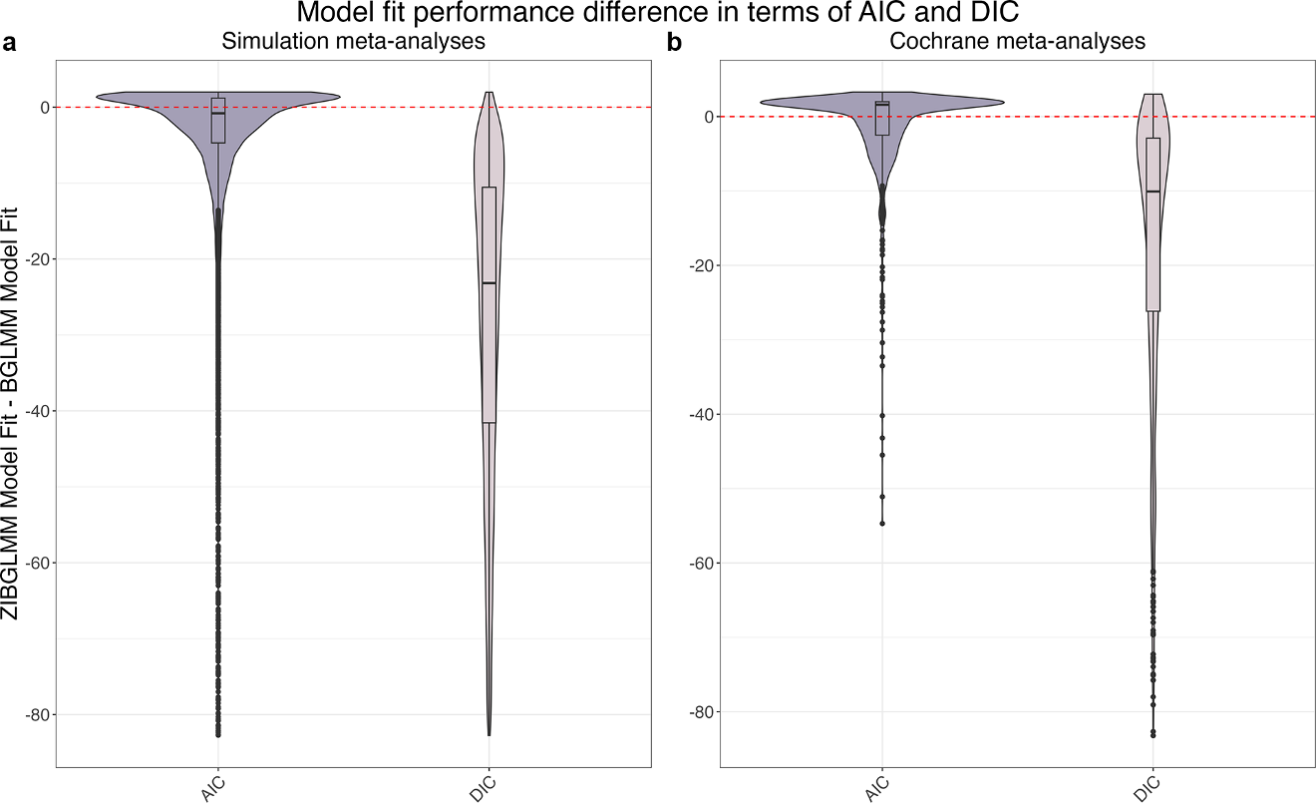
Model goodness of fit in terms of Akaike information criterion (AIC) and deviance information criterion (DIC) differences between the ZIBGLMM and BGLMM methods for simulation meta-analyses (MAs) in subfigure (a) and Cochrane MAs in subfigure (b). Each violin plot^66^ included a box plot where the box limits indicated the range of the central 50% of the data (i.e., the range between the 25th and 75th percentiles), and the median value was marked by a central black line, along with a kernel smoothed density plot representing the probability distribution. The *y*-axis in each subplot represents the goodness of fit of ZIBGLMM subtracting the goodness of fit of BGLMM. The dashed line represents when the difference is 0. Subfigure (a) illustrates the distribution of AIC and DIC differences for 18,000 simulation datasets. The ZIBGLMM exhibited superior fit for 8,839 of 16,215 (54.51%) simulation studies as measured by AIC, and for 17,805 out of 18,000 (98.92%) simulation studies when measured by DIC. Subfigure (b) illustrates the distribution of AIC and DIC differences for 1,111 Cochrane MAs. The ZIBGLMM exhibited superior fit for 365 of 1,010 (36.13%) Cochrane MAs as measured by AIC and for 986 out of 1,111 (88.74%) Cochrane MAs when measured by DIC.

Finally, we compared the frequentist and Bayesian methods of BGLMM and ZIBGLMM in terms of their goodness of fit using AIC and DIC. Figure 3b displays the distribution of AIC and DIC differences for the Cochrane MAs, with the *y*-axis representing the difference in goodness of fit between ZIBGLMM and BGLMM. The violin plot included a box plot where the box limits indicated the range of the central 50% of the data (i.e., the range between the 25th and 75th percentiles), and the median value was marked by a central black line, along with a kernel smoothed density plot representing the probability distribution. ZIBGLMM outperformed BGLMM in 365 of 1,010 (36.13%) Cochrane MAs as measured by AIC, and in 986 of 1,111 (88.74%) datasets based on DIC.

In summary, our meta-meta-analysis study suggests that the inclusion or exclusion of DZS in MAs can influence effect size estimates, with disparities in mean effect size differences reaching up to 0.16. Additionally, both the direction and significance of effect sizes can be altered depending on the methodologies employed. The findings underscore the importance of carefully considering methodological choices in MAs, particularly when handling datasets with double-zero studies.

## 6 Simulations

In addition to the Cochrane datasets, we conducted extensive simulation studies to evaluate our methods in various settings. We simulated MAs with small (10), moderate (25), and large (50) numbers of studies of sample size 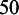 . The nonzero-inflated data were generated using the BGLMM. The number of DZS for each MA was generated from a binomial distribution with proportions of zero-inflation of 25% and 50%. The baseline event risk in the control group was set at 3%, and the average marginal RR was established at 1, 1.5, and 2. Detailed settings of the simulation studies can be found in Table 4. A total of 1,000 MAs were simulated for each of the 18 combinations of settings.

**Table 4:** Specifications for the simulation studies

Parameter	Value
Proportion of zero inflation	25%, 50%
No. of studies	10 (small), 25 (moderate), 50 (large)
Average event probabilities for the nonzero-inflated part	3% for the control group on average, generated from a BGLMM model
Marginal RR	1, 1.5, 2
Study size	50
No. of double-zero studies	Binomial (no. of studies, proportion of zero inflation)

We applied both the frequentist and Bayesian versions of BGLMM and ZIBGLMM to each simulated MA, assessing their goodness of fit with AIC and DIC. The distribution of AIC and DIC differences across the 18,000 simulation datasets is depicted in Figure 3a, with the *y*-axis representing the goodness of fit differences between ZIBGLMM and BGLMM. The violin plot included a box plot where the box limits indicated the range of the central 50% of the data (i.e., the range between the 25th and 75th percentiles), and the median value was marked by a central black line, along with a kernel smoothed density plot representing the probability distribution. ZIBGLMM outperformed BGLMM in 8,839 of 16,215 (54.51%) simulation studies in terms of AIC and in 17,805 of 18,000 (98.92%) studies based on DIC. These findings underscore ZIBGLMM’s robustness in the simulated scenarios.

**Figure 4:**
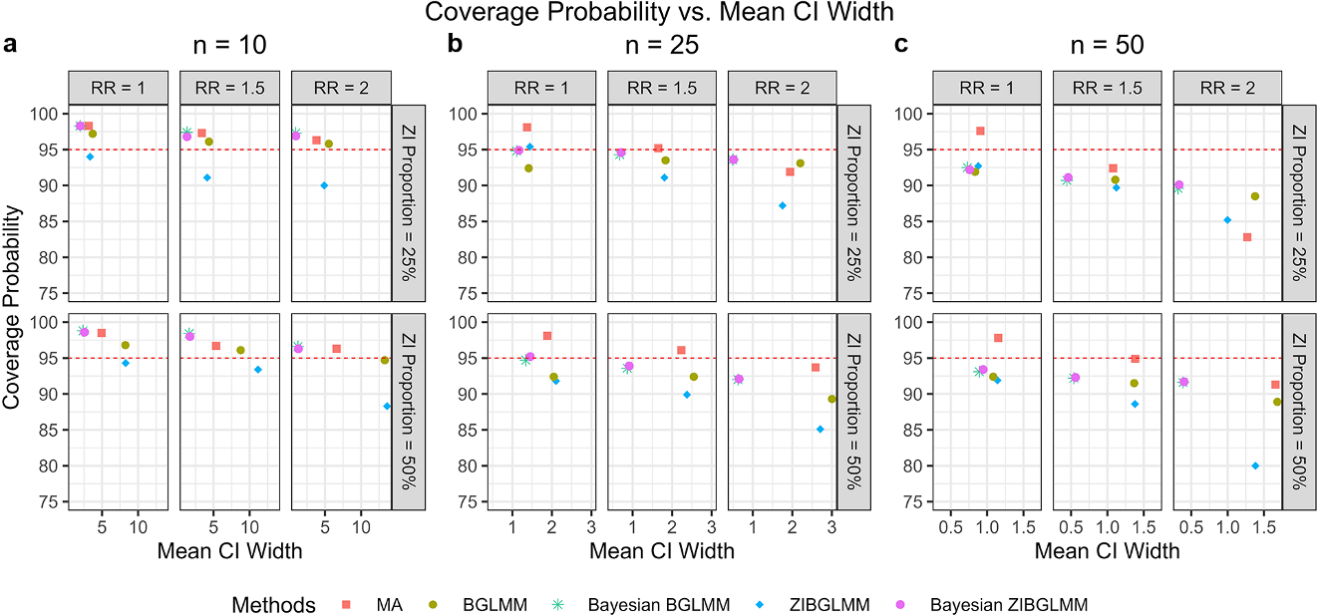
Coverage probability and mean CI length of conventional two-stage meta-analysis (MA), bivariate generalized linear mixed models (BGLMM), Bayesian BGLMM, zero-inflated bivariate generalized linear mixed models (ZIBGLMM), and Bayesian ZIBGLMM for meta-analyses with 10 studies (a), 25 studies (b), and 50 studies (c). The *y*-axis displays the coverage probability, and the *x*-axis displays the mean 95% confidence/credible interval widths. The number of studies in an MA is denoted by *n*. The Bayesian BGLMM and Bayesian ZIBGLMM displayed comparable, consistently high coverage probabilities to MA, while maintaining the shortest mean credible interval widths across all settings. The coverage probabilities for all methods decreased as the average marginal risk ratio increased and as the size of studies increased.

We compared the coverage properties of conventional two-stage MAs excluding DZS and both the frequentist and Bayesian versions of BGLMM and ZIBGLMM. Figure 4 shows the coverage probabilities, along with the mean lengths of confidence intervals (frequentist models) and credible intervals (Bayesian models), while detailed coverage probabilities can be found in Table 5. Remarkably, the Bayesian BGLMM and Bayesian ZIBGLMM display comparable, consistently high coverage probabilities to MA, while maintaining the shortest mean confidence interval widths across all settings among all five methods. The coverage probabilities for all methods decrease as the average marginal RRs increase and as the size of studies increases. The coverage properties of frequentist BGLMM and ZIBGLMM were calculated contingent on the number of MAs that attained convergence.

**Table 5:** Coverage probability of the five methods: two-stage meta-analysis excluding DZS (MA), BGLMM, Bayesian BGLMM, ZIBGLMM, and Bayesian ZIBGLMM

			Frequentist		Bayesian	
			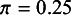	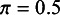	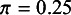	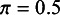
MA	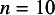	RR 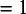	98.3%	98.5%	—	—
		RR 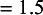	97.3%	96.7%	—	—
		RR 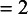	96.3%	96.3%	—	—
	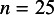	RR 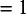	98.1%	98.1%	—	—
		RR 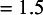	95.2%	96.1%	—	—
		RR 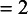	91.9%	93.7%	—	—
	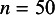	RR 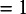	97.6%	97.8%	—	—
		RR 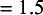	92.4%	94.9%	—	—
		RR 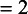	82.8%	91.3%	—	—
BGLMM	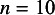	RR 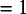	97.2%	96.8%	98.3%	98.8%
		RR 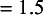	96.1%	96.1%	97.4%	98.4%
		RR 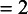	95.8%	94.7%	97.3%	96.6%
	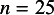	RR 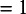	92.4%	92.4%	94.8%	94.7%
		RR 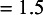	93.5%	92.4%	94.3%	93.6%
		RR 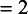	93.1%	89.3%	93.6%	92%
	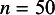	RR 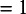	91.9%	92.4%	92.5%	93.1%
		RR 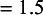	90.8%	91.5%	90.7%	92.2%
		RR 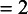	88.5%	88.9%	89.6%	91.6%
ZIBGLMM	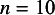	RR 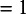	94%	94.3%	98.3%	98.6%
		RR 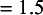	91.1%	93.4%	96.8%	98%
		RR 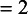	90%	88.3%	96.9%	96.3%
	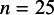	RR 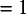	95.4%	91.8%	94.9%	95.2%
		RR 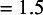	91.1%	89.9%	94.6%	93.9%
		RR 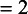	87.2%	85.1%	93.6%	92.1%
	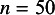	RR 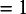	92.7%	91.9%	92.2%	93.4%
		RR 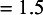	89.7%	88.6%	91.1%	92.3%
		RR 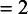	85.2%	80%	90.1%	91.7%

**Figure 5:**
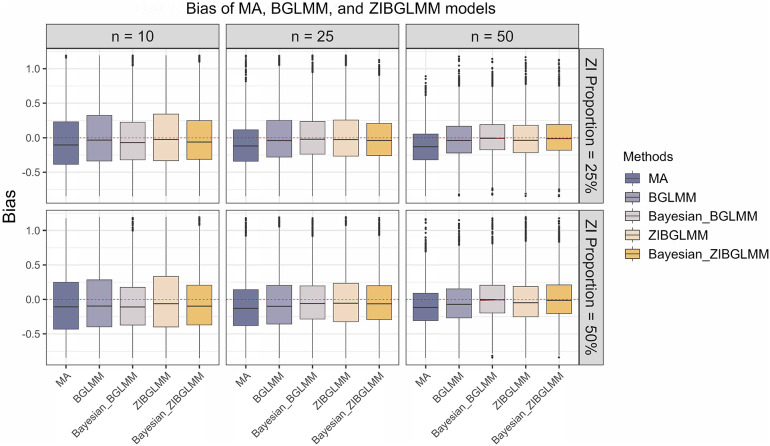
Bias in the estimation of risk ratio from conventional two-stage meta-analysis excluding DZS (MA), alongside the frequentist and Bayesian versions of BGLMM and ZIBGLMM. For meta-analyses (MAs) with a smaller size (10 studies), the frequentist ZIBGLMM exhibits the least bias. For MAs with a moderate size (25 studies), Bayesian BGLMM manifests the least bias. The Bayesian BGLMM and ZIBGLMM archives the smallest bias for large (50 studies) MAs. Frequentist ZIBGLMM consistently demonstrates less bias than the frequentist BGLMM. For all settings, both frequentist and Bayesian BGLMM and ZIBGLMM consistently yield smaller biases compared to MA.

Figure 5 shows the bias in the estimation of RR obtained from conventional two-stage MA excluding DZS, along with the frequentist and Bayesian versions of BGLMM and ZIBGLMM. The frequentist ZIBGLMM obtains the least bias for MAs of small size (10 studies), while the Bayesian BGLMM manifests the least bias for MAs of moderate size (e.g., with 25 studies) among the five methods. The Bayesian BGLMM and ZIBGLMM obtain effect size estimates with a comparatively small bias for large MAs (e.g., with 50 studies).

Moreover, the frequentist ZIBGLMM consistently demonstrates less bias than the frequentist BGLMM. For all settings, both frequentist and Bayesian BGLMM and ZIBGLMM consistently yielded smaller biases compared to conventional two-stage MAs that exclude DZS. We observe an increased number of convergence issues for frequentist ZIBGLMM, especially for smaller MAs with 10 studies. Possible contributing factors could be the absence of DZS or an excessive presence of DZS within an MA. Comprehensive details on the convergence issues for *n* = 10 can be found in Table 6 in the Supplementary Material. This convergence issue by the zero-inflated models is consistent with the observations made by Beisemann et al.^31^

**Figure 6:**
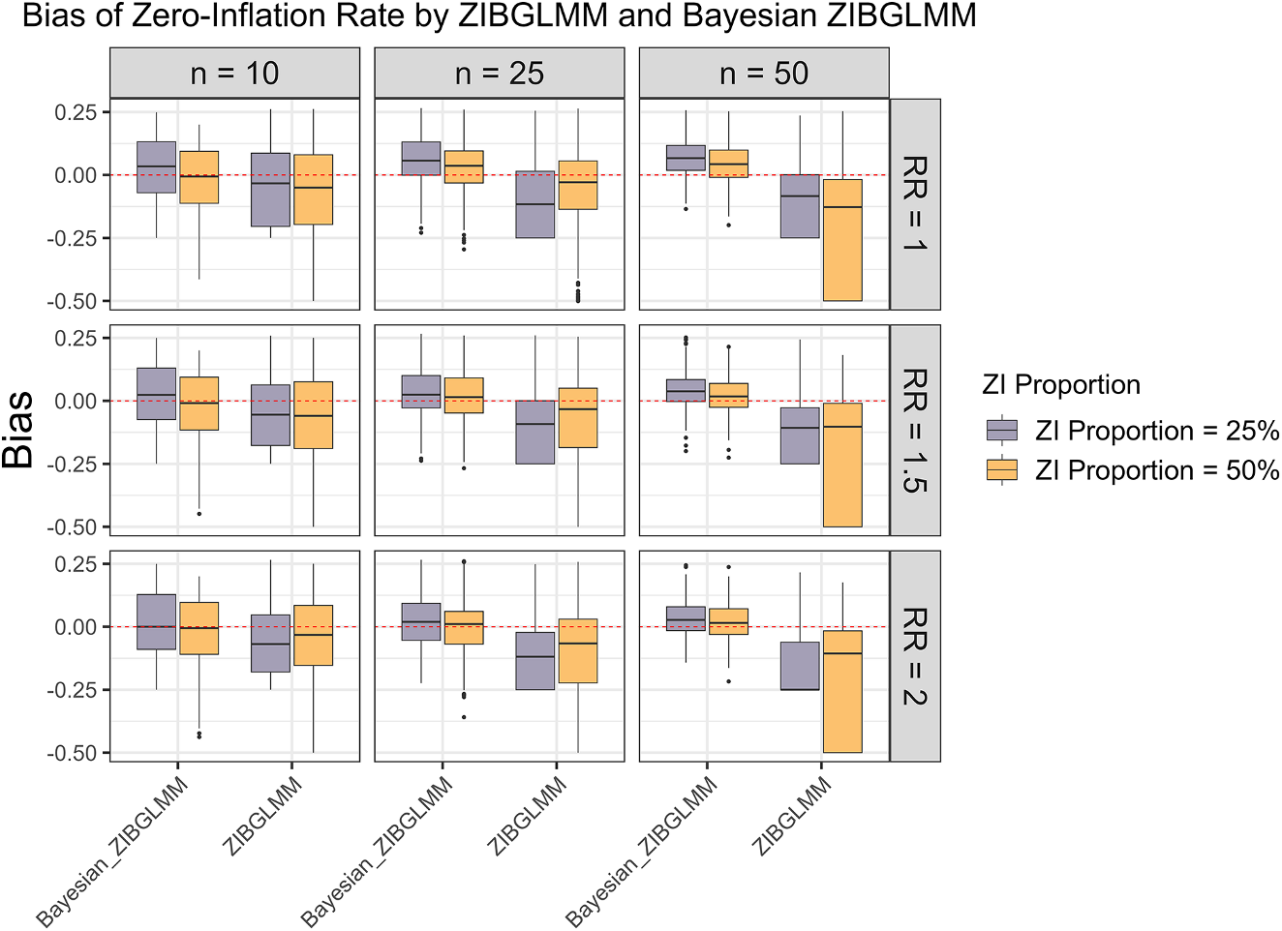
Bias in the estimation of proportion of zero inflation 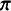 . Bayesian ZIBGLMM produces an estimate of the proportion of zero inflation with a smaller bias than the frequentist ZIBGLMM across all study sizes and proportions of zero inflation. However, it tends to overestimate the proportion of zero inflation for larger meta-analyses (MAs) (50 studies). In addition, Bayesian ZIBGLMM yields better estimates when the proportion of zero inflation is 50% than when it is 25%. The frequentist ZIBGLMM appears to have a more substantial bias as the size of the MAs grows.

An important advantage of the proposed ZIBGLMM method is its capability of capturing population heterogeneity by estimating the proportion of zero inflation. To evaluate the empirical performance of ZIBGLMM under various settings, Figure 6 summarizes the bias of the estimated proportion of zero inflation with respect to the frequentist and Bayesian ZIBGLMM methods. We observe that Bayesian ZIBGLMM produces estimates of the proportion of zero inflation with smaller biases than the frequentist ZIBGLMM across MAs of all sizes and proportions of zero inflation. In addition, Bayesian ZIBGLMM yields more accurate estimates when the proportion of zero inflation is 50% than when the proportion of zero-inflation is 25%. In contrast, the frequentist ZIBGLMM appears to have a more substantial bias as the size of the MAs grows.

In summary, our extensive simulation studies suggest that ZIBGLMM, especially the Bayesian method, is a promising tool for MAs, particularly when dealing with datasets that have varying proportions of DZS. This method provides robust model fit, smaller bias in effect size estimation, and comparatively high coverage probabilities as conventional two-stage MA. Moreover, the capability of Bayesian ZIBGLMM to estimate the proportion of zero inflation accurately, especially in smaller and moderate-sized MAs, further emphasizes its significance in capturing the underlying population heterogeneity. However, practitioners need to be cautious while interpreting results from larger MAs, as there may be a tendency to overestimate the proportion of zero-inflation. It is also worth noting the potential convergence issues faced by the frequentist ZIBGLMM method in certain scenarios, which might be solved by using different optimization algorithms.

## 7 Discussion

In this article, we proposed a ZIBGLMM as a new method for handling DZS in MA. This model is motivated by the hypothesis that zeros in DZS may arise due to heterogeneity in the population, making the segregation of “structural” and “chance” zeros essential for accurate analysis.

The main estimate that we derive from our model is a marginal risk ratio (RR). Alternatively, one could use the OR as the estimate. We chose RR over OR as the estimate mainly because of the non-collapsibility issues of the OR. There has been an ongoing debate on the choice between OR and RR.^56^ ^–^ ^59^ Another option is to use a GLMM with a log link assuming a Poisson distribution within studies as Böhning et al.^30^ to report cluster-specific effects instead of marginal effects.^60^ The choice between reporting the cluster-specific effects versus the marginal effects depends on the population we are interested in generalizing to. Marginal effects should be used if the goal is to generalize to the whole population, while cluster-specific effects should be used if we are only interested in the study-level effects. One potential issue with the bivariate approach to MA is that it may permit intertrial information to be recovered, which in theory can lead to bias.^61^ However, as Senn^61^ has pointed out, in practice, the size of this bias is likely to be small and can rarely cause an issue in real-world applications.

There are two ways of defining the treatment effects in our model setting. Let 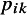 denote the probability of success for the *i*th study for the *k*th treatment arm. Then, we could estimate the treatment effects based on the overall population (both ZI and non-ZI population), and the formula is 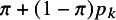 , where 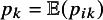 . The argument for this approach is that this aligns more with our model of the two mixture parts or only the non-ZI population, and the formula is 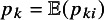 . The argument for this is that since the population is ZI and is healthier, then there are little treatment effects for that population. We have chosen the latter way of defining the treatment effects.

Using 1,111 real-world MAs with DZS selected from the CDSR and 18,000 simulated MAs, we demonstrated the strengths and potential benefits of both the frequentist and Bayesian versions of ZIBGLMM. The Bayesian ZIBGLMM provided consistently better goodness of fit than the Bayesian BGLMM across varying scenarios. We further identified that Bayesian BGLMM and Bayesian ZIBGLMM generally accomplished comparable coverage probabilities to two-stage MAs, shorter confidence interval lengths, and less bias in estimating RR.

The ZIBGLMM method has several limitations compared with the standard bivariate generalized linear model or conventional two-stage MA excluding DZS. Notably, convergence issues might occur for the frequentist versions of ZIBGLMM and BGLMM models, especially for MAs with a smaller number of studies. This issue potentially stems from the absence of DZS or the excessive presence of DZS in an MA. The frequentist ZIBGLMM method has a higher chance of encountering convergence issues than the BGLMM method. Specifically, 1,015 datasets converged for ZIBGLMM, while 1,084 datasets converged for BGLMM for the 1,111 CDSR MAs. A potential way of avoiding convergence issues is to change the optimization algorithms used. Alternatively, using the Bayesian BGLMM and ZIBGLMM can avoid the convergence issues.

The limitation of the Bayesian BGLMM and ZIBGLMM is that they typically might take longer time to compute than the frequentist counterparts. For example, fitting a Bayesian ZIBGLMM on an MA with 25 studies takes around 4 minutes, and fitting on an MA with 50 studies takes around 9 minutes on a MacBook Pro with an Apple M1 Pro 10-core CPU and 32 GB RAM.

Our general recommendation to practitioners is to opt for Bayesian ZIBGLMM in the presence of DZS, as it provides a more accurate estimation of the RR and the proportion of zero inflation. Bayesian ZIBGLMM in general achieves better goodness of fit in terms of deviance of information (DIC) than Bayesian BGLMM. In addition, the Bayesian methods are not subject to convergence issues that the frequentist ZIBGLMM might face. The main limitation of the Bayesian methods is that the computation time might be longer for a single MA than their frequentist counterparts.

If computation time is a major limitation, we recommend using the frequentist ZIBGLMM, which can provide a more accurate estimation of the RR than the frequentist BGLMM. For MAs comprising 10 or fewer studies, we recommend using Bayesian ZIBGLMM for MAs with 10 studies or fewer since the computation time would not increase too much and the frequentist ZIBGLMM are more likely to experience convergence for the small MAs as observed in the simulation studies. We also recommend exercising caution with large MAs when using Bayesian ZIBGLMM, as there might be a tendency to overestimate the proportion of zero inflation.

We note that when the true data-generating mechanism is BGLMM rather than ZIBGLMM, using ZIBGLMM might be misspecified. In fact, the BGLMM is a submodel of the ZIBGLMM. In practice, if there are excessive numbers of zeros in the data, we recommend the investigators to fit the ZIBGLMM to avoid substantial biases due to underfitting. Further, we can also formally test between the BGLMM and ZIBGLMM models, as they are nested models. Score tests and LR tests can be developed following the work by Hall et al.^62^ and Huang et al.^63^ For the LR test, one needs to properly account for the fact that under the null hypothesis, the mixing probability parameter 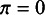 lies at the boundary of its parameter space [0, 1].^41^ ^,^ ^64^ ^,^ ^65^

Meta-regression remains a challenging yet vital aspect of enhancing the robustness and applicability of our findings. In particular, when many zero events occur in MAs, the effect sizes tend to be more homogeneous, making it difficult to identify appropriate study-level summary covariates that could explain small between-study heterogeneity. One direction for future work is to expand the current model to incorporate covariate meta-regression, which would allow for a more detailed examination of how specific study characteristics influence the treatment effects.

In conclusion, our study proposes the ZIBGLMM as a novel approach to handling DZS, which can effectively reduce the biases in conventional MA methods by properly accounting for the between-population heterogeneity. We expect the ZIBGLMM method to be useful in pharmacoepidemiological and pharmacovigilance studies where the event probabilities are rare and DZS are prevalent.

## Supporting information

Li et al. supplementary material Li et al. supplementary material

## Data Availability

The datasets and code for this study can be found in the repository.^32^ DOI: https://doi.org/10.5281/zenodo.14827287.

## Supplementary material

The supplementary material for this article can be found at https://doi.org/10.1017/rsm.2024.4.
