# Supplementary material for: ZIBGLMM: Zero-inflated bivariate generalized linear mixed model for meta-analysis with double-zero-event studies
Source: Res Synth Methods. 2025 Mar 21;16(2):251–71. doi: 10.1017/rsm.2024.4 (PMC12527523; doi:10.1017/rsm.2024.4)
Supplement: Li et al. supplementary material [file S1759287924000048sup001.pdf]

Supplementary Material

Table 1: Summary of the meta-analyses that converged for the frequentist BGLMM and ZIBGLMM methods in the simulation study. π denotes the proportion of zero-inflation, n is the number of studies in the simulated meta-analysis, and RR is the true marginal risk ratio. "# covered" is the number of meta-analyses whose confidence intervals contain the true risk ratio. "# is not NA" is the number of meta-analyses whose random effect and fixed effect for both treatment arms are available using the frequentist methods. "# of converged" is the total number of meta-analyses for which the frequentist methods converged (i.e., the model successfully executes without encountering the "Optimization cannot be completed" error). All the numbers are out of a total of 1,000 simulated meta-analyses.

				# covered	# not NA	# of converged
BGLMM	π = 0.25	n=10	RR=1	847	871	977
			RR=1.5	819	852	977
			RR=2	820	856	984
		n=25	RR=1	727	787	952
			RR=1.5	809	865	979
			RR=2	825	886	980
		n=50	RR=1	834	908	981
			RR=1.5	853	939	991
			RR=2	852	963	999
	π = 0.5	n=10	RR=1	847	875	981
			RR=1.5	799	831	970
			RR=2	815	861	974
		n=25	RR=1	746	807	958
			RR=1.5	789	854	977
			RR=2	781	875	983
		n=50	RR=1	843	912	975
			RR=1.5	866	946	987
			RR=2	849	955	993
ZIBGLMM	π = 0.25	n=10	RR=1	772	821	932
			RR=1.5	703	772	890
			RR=2	668	742	860
		n=25	RR=1	730	765	953
			RR=1.5	713	783	917
			RR=2	635	728	868
		n=50	RR=1	728	785	939
			RR=1.5	777	866	955
			RR=2	662	777	874
	π = 0.5	n=10	RR=1	788	836	920
			RR=1.5	735	787	881
			RR=2	651	737	830
		n=25	RR=1	738	804	898
			RR=1.5	683	760	875
			RR=2	549	645	783
		n=50	RR=1	718	781	922
			RR=1.5	697	787	906
			RR=2	517	646	760
